# Transgenic Expression of Human *LAMA5* Suppresses Murine *Lama5* mRNA and Laminin α5 Protein Deposition

**DOI:** 10.1371/journal.pone.0023926

**Published:** 2011-09-07

**Authors:** Brooke M. Steenhard, Adrian Zelenchuk, Larysa Stroganova, Kathryn Isom, Patricia L. St. John, Glen K. Andrews, Kenneth R. Peterson, Dale R. Abrahamson

**Affiliations:** 1 Departments of Anatomy and Cell Biology, University of Kansas Medical Center, Kansas City, Kansas, United States of America; 2 Biochemistry and Molecular Biology, University of Kansas Medical Center, Kansas City, Kansas, United States of America; 3 The Kidney Institute, University of Kansas Medical Center, Kansas City, Kansas, United States of America; Institut National de la Santé et de la Recherche Médicale, France

## Abstract

Laminin α5 is required for kidney glomerular basement membrane (GBM) assembly, and mice with targeted deletions of the *Lama5* gene fail to form glomeruli. As a tool to begin to understand factors regulating the expression of the *LAMA5* gene, we generated transgenic mice carrying the human *LAMA5* locus in a bacterial artificial chromosome. These mice deposited human laminin α5 protein into basement membranes in heart, liver, spleen and kidney. Here, we characterized two lines of transgenics; Line 13 expressed ∼6 times more *LAMA5* than Line 25. Mice from both lines were healthy, and kidney function and morphology were normal. Examination of developing glomeruli from fetal *LAMA5* transgenics showed that the human transgene was expressed at the correct stage of glomerular development, and deposited into the nascent GBM simultaneously with mouse laminin α5. Expression of human *LAMA5* did not affect the timing of the mouse laminin α1–α5 isoform switch, or that for mouse laminin β1–β2. Immunoelectron microscopy showed that human laminin α5 originated in both glomerular endothelial cells and podocytes, known to be origins for mouse laminin α5 normally. Notably, in neonatal transgenics expressing the highest levels of human *LAMA5*, there was a striking reduction of mouse laminin α5 protein in kidney basement membranes compared to wildtype, and significantly lower levels of mouse *Lama5* mRNA. This suggests the presence in kidney of a laminin expression monitor, which may be important for regulating the overall production of basement membrane protein.

## Introduction

The human kidney contains approximately one million individual nephrons, each beginning with a glomerulus, which is a unique capillary tuft that largely restricts the passage of serum albumin and larger proteins into the primary nephron filtrate. All three layers of the glomerular capillary wall, namely the glomerular endothelial cells, glomerular epithelial cells (podocytes), and an intervening glomerular basement membrane (GBM), are required for maintenance of normal filtration barrier properties [1]–[3]. For example, enzymatic degradation of glycosaminoglycans within the glomerular endothelial surface glycocalyx results in an increased fractional clearance for albumin [4]. Additionally, blockage of podocyte-derived VEGF signaling causes glomerular endothelial cell abnormalities in developing or mature kidneys and proteinuria [5], [6].

A host of defects that affect the podocyte and its specialized intercellular junction, the epithelial slit diaphragm, also cause abnormal glomerular permeabilities [1]–[3]. These include mutations in the *NPHS1* gene encoding the slit diaphragm component, nephrin, which causes congenital nephrotic syndrome of the Finnish type and results in massive proteinuria at birth [7]. Mutations to *NPHS2*, which encodes another slit diaphragm protein, podocin, also causes proteinuria in autosomal recessive steroid-resistant nephrotic syndrome, a disease often diagnosed in childhood [8]. Intracellularly, podocin and nephrin are both linked indirectly to the actin cytoskeleton through interaction with CD2-associated protein (CD2ap) [9], [10]. Slit diaphragms are absent in mice that lack nephrin [11] or podocin [12] and these animals die perinatally with renal failure. Mice deficient in CD2ap also die from renal failure, but at 6–7 weeks of age [10].

Like all basement membranes, the GBM is composed of type IV collagen, laminin, nidogen, and proteoglycans [13]. Unlike most other basement membranes, however, the GBM undergoes type IV collagen and laminin isoform substitution during glomerular development [14], [15]. Specifically, basement membranes within the earliest glomerular regions of comma- and S-shaped nephric figures contain collagen α1α2α1(IV) and laminin α1β1γ1 (LN-111). These isoforms are later replaced by collagen α3α4α5(IV), laminin α5β1γ1 (LN-511), and laminin α5β2γ1 (LN-521) as glomerular capillary loops expand. Subsequently, LN-521 is the only laminin isoform found in GBMs of fully mature glomeruli. Collagen α1α2α1(IV) and all of the GBM laminin chains originate from both glomerular endothelial cells and podocytes [16], [17], but collagen α3α4α5(IV) derives solely from podocytes [17].

The reasons why the GBM collagen IV and laminin composition changes during development are not fully understood, but evidence indicates that this is necessary for final glomerular maturation and full acquisition and maintenance of filtration barrier properties. Alport disease, which is a familial nephropathy marked by focal splitting, thinning, and regional thickening of the GBM and leads to renal failure, is caused by mutations in either the *COL4A3*, *COL4A4*, or *COL4A5* genes encoding the collagen α3(IV), α4(IV), and α5(IV) protein chains, respectively [18], [19]. Most Alport patients fail to assemble a stable network of collagen α3α4α5(IV) in the GBM, and there is retention of the infantile, collagen α1α2α1(IV) network. This isoform appears to be more susceptible to proteolysis, which may explain why the GBMs of Alport patients ultimately deteriorate [19]. A model of Alport disease has been created in mice through the deletion of the *Col4a3* gene [20]–[22], and these animals die of renal failure 2–4 months after birth with the same glomerular defects as those seen in Alport patients. The mouse Alport phenotype can be rescued when transgenic mice expressing human *COL4A3-COL4A4* genes are crossed onto the mouse *Col4a3* knockout background [23].

Failure to undergo laminin isoform transitioning from LN-111 to LN-521 also results in kidney malfunction in mice and in humans. Although normal glomerular development is seen in mice with laminin β2 deficiencies, they eventually exhibit podocyte foot process broadening, proteinuria, and die of renal failure [24]. Humans with mutations in the *LAMB2* gene suffer from Pierson syndrome, which usually presents at birth as congenital nephrotic syndrome with severe neuromuscular junction abnormalities (owing to the presence of laminin β2 in the neuromuscular junction basement membrane as well) [25].

There are no human mutations described for *LAMA5*, but experiments in mice have shown its expression to be absolutely crucial for normal glomerular development and function. Mice with deletions of *Lama5* die before birth with neural tube closure defects and placental dysmorphogenesis [26]. In kidney, a stable GBM fails to assemble, and endothelial cells do not form vascularized glomerular tufts [27]. This *Lama5* knockout phenotype can be partially rescued when fetal kidneys from *Lama5* mutants are grafted into newborn kidneys of normal, wildtype hosts [28]. In this case, host endothelial cells, which express laminin α5, migrate into the engrafted *Lama5* null kidneys and vascularized glomeruli form within grafts. The host endothelial cell-derived laminin α5 does not project across the full width of these GBMs, however. This results in an unusual situation where there is retention of the infantile laminin α1 on the outer, sub-podocyte layer of matrix and laminin α5 is present only on the inner, subendothelial layer. Additionally, these hybrid GBMs are abnormally wide and not as well condensed as normal GBM, and podocyte foot processes are absent [28]. In other experiments, deletion of *Lama5* only in podocytes results in mild to severe proteinuria, and variable defects in GBM and podocyte ultrastructure [29]. In this same study, expression of a human *LAMA5* transgene under control of a doxycyclin inducible, podocyte-specific expression system rescues glomerular and tubular defects caused by a hypomorphic *Lama5* mutation [29].

Taken together, these findings demonstrate that the timely expression of LN-521 is needed for glomerular endothelial cell and podocyte differentiation, and the appearance of collagen α3α4α5(IV) is required for long term GBM stability. However, very little is known at the gene level regarding activation of any of the mature GBM protein isoforms, what silences transcription of infantile chain genes at the appropriate developmental stages, and how the infantile collagen α1α2α1(IV) and LN-111 networks are removed from developing GBM. In addition, we do not understand what causes upregulation of *Lama5* in *Col4a3* knockout (Alport) mice, which may be an important contributor to fibrosis in that model [30].

To begin addressing some of these questions, we have developed bacterial artificial chromosome (BAC) transgenic mice expressing human *LAMA5*. These transgenics deposited apparently large amounts of human laminin α5 protein in basement membranes widely, and, specifically in glomeruli, at the appropriate developmental stage. Expression of human *LAMA5* did not appear harmful and kidney functional tests and morphology were normal. The results suggest that the BAC used for transgenic injections contained all of the necessary regulatory information for proper *LAMA5* expression. Of great interest, in kidneys from lines with the highest levels of human *LAMA5* expression, there were significant decreases in native mouse *Lama5* mRNA and mouse laminin α5 protein deposition.

## Materials and Methods

### Generation of human chromsome 20 BAC transgenics

All experiments with mice strictly followed policies and procedures established by the Animal Welfare Act and the Public Health Service Policy on the Humane Care and Use of Laboratory Animals. Human BAC clone RP11-1023E23, containing ∼189 kB of human chromosome 20, was obtained from Empire Genomics (Buffalo, NY). Bacteria were grown in 15 µg/ml chloramphenicol overnight at 33°C, then BAC DNA was purified using a Nucleobond kit (Clontech, Mountain View, CA). The clone was verified by end-sequencing, polymerase chain reaction and restriction enzyme digestion followed by pulsed field gel electrophoresis. DNA was injected into FvB×C57Bl/6 F1 hybrid oocytes by personnel from the Transgenic and Gene Targeting Institutional Facility at the University of Kansas Medical Center. To screen founders, human-specific primers were designed to intronic regions of all five genes residing along the BAC clone: *OSBPL2*, *ADRM1*, *LAMA5*, *RPS21* and *C20orf151* using the Roche Universal Probe Library (Roche Applied Science, Indianapolis, IN). Primers were tested for specificity using mouse and human genomic DNA (Table S1). Genomic DNA from each founder was purified using Qiagen tissue kits (Valencia, CA). Founders that amplified all 5 human gene products were mated to wildtype C57Bl/6 mice, and several stable lines were established. Colonies from two of these lines, which expressed high and moderate levels of human *LAMA5* (Lines 13 and 25, respectively), were maintained by mating BAC transgenic heterozygotes with wildtype C57Bl/6 mice.

### Verification of human *LAMA5* expression

Transgenic N1 BAC progeny, at various ages, were killed and heart, liver, spleen and kidney tissues were promptly harvested, surrounded by Tissue Tek O.C.T. Compound (Electron Microscopy Sciences, Fort Washington, PA), and frozen immediately in isopentane chilled in a dry ice-acetone bath. Cryostat sections, five µm thick, were labeled with mouse anti-human laminin α5 antibody (1∶500 dilution, clone 4C7, Millipore, Billerica, MA) or rabbit anti-mouse laminin α5 antibody (1∶200 dilution; antibody was a kind gift from Dr Jeffery Miner, Washington University, St Louis, MO). Appropriate, species specific secondary antibodies conjugated to Alexa Fluor dyes were used at a 1∶200 dilution (Invitrogen/Molecular Probes, Eugene, OR). In some cases, sections were dually labeled with monoclonal rat anti-mouse laminin α1 or β1 IgGs (50 µg/ml) [31] and rabbit anti-mouse laminin α5 (1∶200) or rabbit anti-mouse laminin β2 (1∶2,000) (from Dr. Jeffery Miner). Slides were mounted using Prolong Gold plus DAPI (Molecular Probes). Sections were viewed and imaged by standard epifluorescence on a Leica SM5000B microscope (Bannockburn, IL). Slides were also examined with a Zeiss LSM 510 scanning laser confocal microscope (Thornwood, NY) and images were captured at an optical section thickness of 0.2 µm.

### qPCR

At the time of sacrifice, a portion of kidney tissue was collected in RNAlater (Qiagen) and stored at −80°C. Total RNA was purified using a RNeasy Mini kit (Qiagen), incubated with primers designed to hybridize specifically with human LAMA5, mouse Lama5, Lamb1, and Lamc1 RNA (Table S1) and Quantitect SYBR Green RT-PCR reagents. Products were amplified and quantified in an iCycler (BioRad, Hercules, CA). Relative RNA abundance was determined using the comparative Ct method [32]. Normal human kidney total RNA served as a reference and was purchased from Clontech Laboratories, Inc. (Mountain View, CA).

### Estimate of BAC LAMA5 copy number

A standard curve was prepared containing between 1 and 128 copies of human BAC clone RP11-1023E23 in 5 nanograms of wildtype mouse genomic DNA. Genomic DNA was isolated from transgenic liver tissue from both Line 13 and Line 25 using DNeasy Blood and Tissue kit (Qiagen). The standard curve and genomic DNA samples were amplified with human LAMA5 genomic primers and mouse hemoglobin alpha primers (Table S1) with the RT^2^ SYBR Green/Fluorescein qPCR Master Mix (SABiosciences, Frederick, MD). Relative abundance was estimated using the comparative Ct method [32].

### Blood and urine chemistries

Blood urea nitrogen was measured from serum samples using the QuantiChrom Urea Assay kit (BioAsssay Systems, Hayward, CA). Urinary albumin was measured using an enzyme-linked immunosorbent assay mouse Albuwell kit (Exocell, Philadelphia, PA). In some cases, urine was first resolved on polyacrylamide gels, which were then stained with Coomassie Blue.

### Electron microscopy

For routine electron microscopy, kidneys were fixed and processed as described [33] and imaged on a JEOL JEM-1400 transmission electron microscope. For postfixation immunoelectron microscopy, 2-mm wedges of kidney cortices were fixed with 1% paraformaldehyde and 0.05% glutaraldehyde in 0.1 M sodium phosphate buffer, pH 7.3, for 1.5 hours on ice. Tissues were washed, equilibrated with 30% sucrose in buffer, and snap frozen in tissue freezing medium (Triangle Biomedical Sciences, Durham, NC) by using isopentane chilled in a dry ice-acetone bath. Frozen sections (30 µm thick) were collected on Thermanox coverslips (Miles Laboratories, Inc., Naperville, IL), and then air dried at room temperature. Sections were blocked for 30 minutes each in 0.5 M ammonium chloride in PBS and then with 5% goat serum and 0.1% bovine serum albumin in PBS. Sections were then immunolabeled with anti-human laminin α5 clone 4C7 ascites fluid (diluted 1∶50 in PBS) for 1 hour, and washed with PBS. Sections were then incubated with rabbit anti-mouse IgG_2a_-HRP (MP Biomedicals, Solon, OH; 50 µg/ml) for 1 hour, washed, refixed in Karnovsky's fixative, developed for peroxidase histochemistry, and processed for electron microscopy as described previously [17].

### Immunoprecipitation and Western blotting

Kidneys were harvested from postnatal day 0–4 (P0-4) mice (human BAC *LAMA5* Line 13 transgenic and wildtype littermate controls), frozen in liquid nitrogen, and stored at −80°C. Kidneys were dounce homogenized in lysis buffer (10 mM Tris, pH 7.5, 150 mM NaCl, 20 mM beta-glycerol phosphate, 5 mM ethlyenediaminetetraacetic acid, 10% glycerol, 2 mM sodium fluoride, 0.5% Igepal, 1× protease inhibitor cocktail [Sigma-Aldrich, St Louis, MO]), sheared five times by passage through a syringe fitted with a 21 gauge needle, extracted in lysis buffer for 2 hours at 4°C, and centrifuged 10 minutes, 14,000 g at 4°C. Protein content of the supernatant was determined using a DC Protein Assay Kit I (BioRad, Hercules, CA), and the concentration was adjusted to 2.2 µg/µl with lysis buffer. Ten microliters of anti-human laminin α5 mouse monoclonal 4C7 IgG was incubated overnight with the supernatant. To recover 4C7, Fastflow protein G sepharose beads (Protein G Sepharose Fastflow, GE Healthcare, Piscataway, NJ) were added and the solutions were gently rotated for 4 hours at 4°C. Beads were then washed three times with lysis buffer, and three times with tris-buffered saline. Beads were boiled in 2× SDS sample buffer (containing dithiothreitol) and stored at −80°C.

The eluted material was electrophoresed in 5% polyacrylamide gels and transferred to polyvinylidene fluoride membranes. Membranes were probed with rat anti-mouse laminin γ1 (ab17792, Abcam, Cambridge, UK) and rat anti-mouse laminin β1 (clone 5A2) [31] IgGs using Super Signal Western Blot Enhancer (Thermo Fisher Scientific, Rockford, IL). Following incubation with anti-rat IgG-HRP secondary antibodies (GE Healthcare), blots were developed with Pierce chemiluminescence detection (Thermo Fisher Scientific).

## Results and Discussion

### Identification of human *LAMA5* BAC transgenics

Transgenic mice were established from 25 progeny born from zygotes injected with BAC DNA RP11-1023E23 (position 60273754–60463260 of human chromosome 20). Genomic DNA was screened with human-specific primers designed to intronic regions of the five genes found in this region of human chromosome 20 (Table S1). Here we characterize two stable transgenic lines that expressed all five human genes, indicating integration and germ line transmission of the complete BAC DNA.

**Figure 1:**
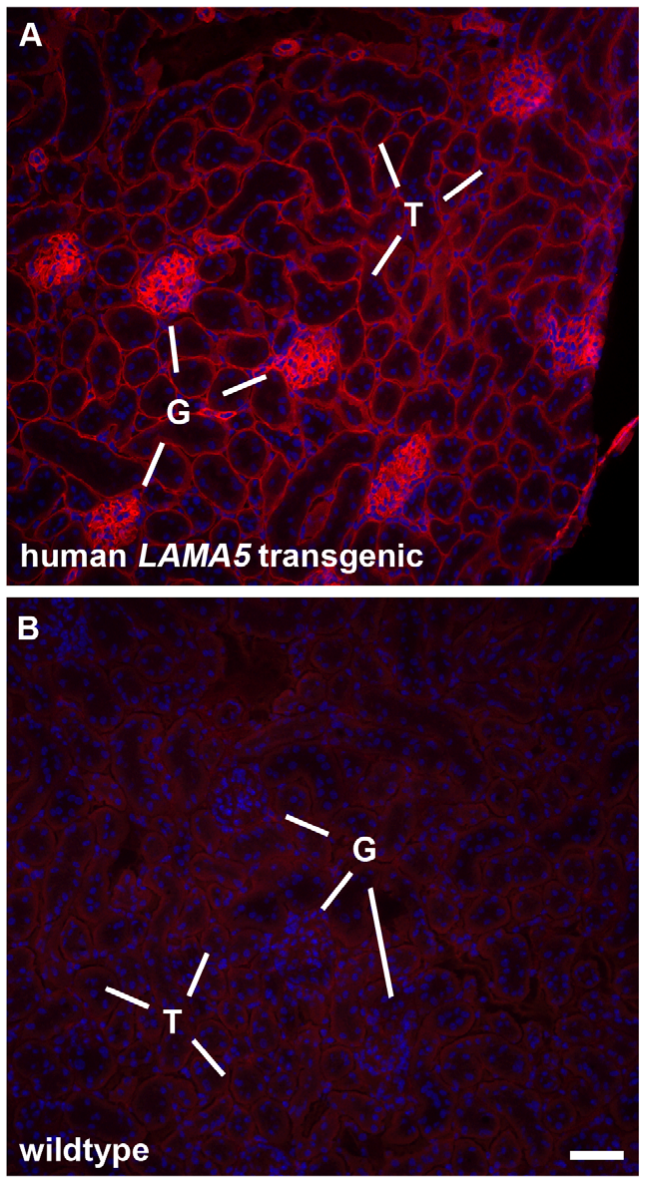
Human *LAMA5* BAC transgenic mice deposit human laminin α5 protein in kidney basement membranes. A: Immunofluorescence micrograph of frozen kidney section from a transgenic mouse labeled with mouse anti-human laminin α5, followed by goat anti-mouse IgG_2a_ conjugated to Alexa Fluor-594. Basement membranes within glomeruli (G) and surrounding tubules (T) immunolabel in bright linear patterns. B: Section from a wildtype littermate incubated with anti-human laminin α5 is negative. In both A and B, cell nuclei are stained blue by DAPI. Magnification: 200×; scale bar = 50 µm.

**Figure 2:**
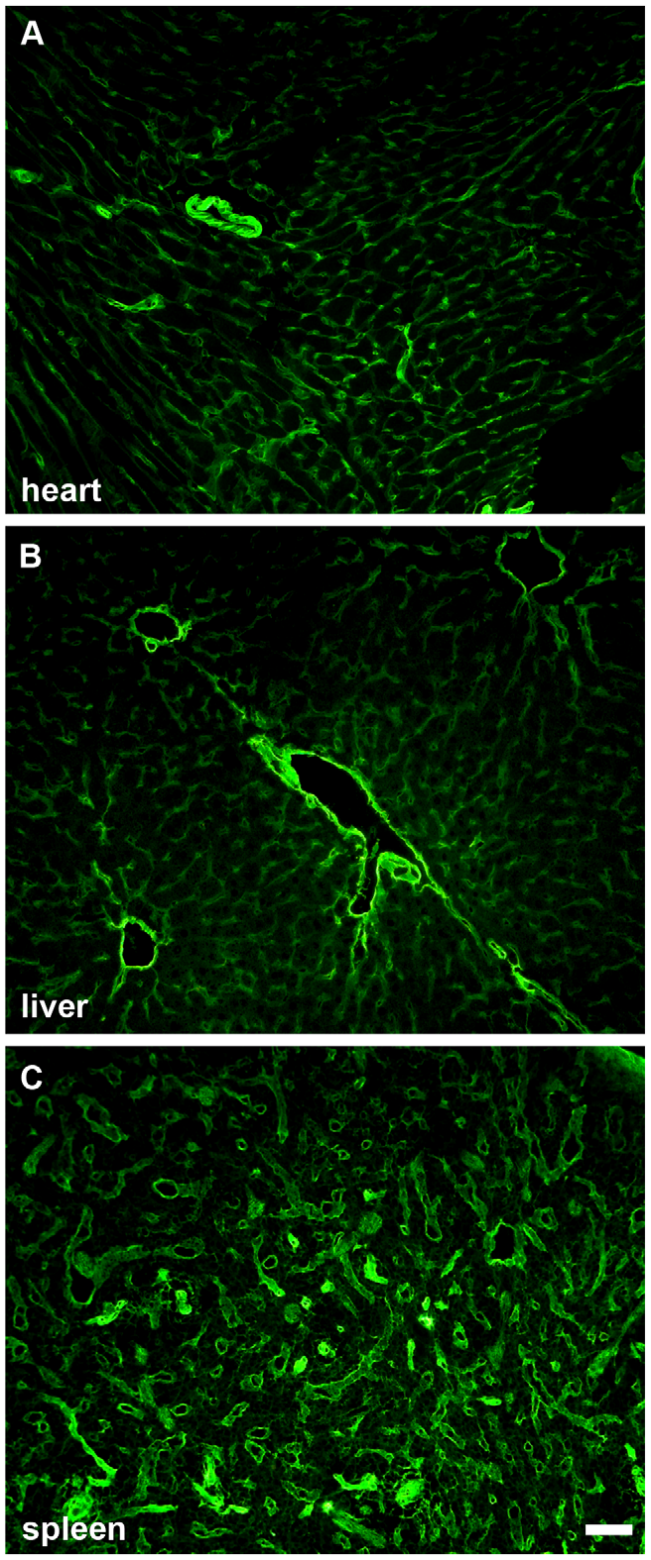
Human *LAMA5* BAC transgenic mice deposit human laminin α5 protein in basement membranes. Frozen sections of tissues from human *LAMA5* BAC transgenics immunolabeled with mouse anti-human laminin α5 antibody and goat anti- mouse IgG_2a_ conjugated to Alexa Fluor-488. Human laminin α5 is deposited widely in most or all basement membranes of heart (A), liver (B), and spleen (C). Magnification: 200×; scale bar = 50 µm.

To confirm transcription and translation of the human *LAMA5* gene in the BAC transgenic mice, frozen sections of kidney from transgenic and wildtype siblings were incubated with monoclonal mouse anti-human laminin α5 IgG and fluorochrome-conjugated secondary antibody. As shown in Figure 1, glomerular and tubular basement membranes from the BAC transgenics were immunolabeled in a bright, linear pattern (Fig. 1A), whereas there was no labeling of sections from wildtype controls (Fig. 1B). In addition, sections of heart, liver, and spleen from BAC transgenics all demonstrated linear basement membrane labeling with anti-human laminin α5 (Figs. 2A–C).

### Quantification of human *LAMA5* expression

**Figure 3:**
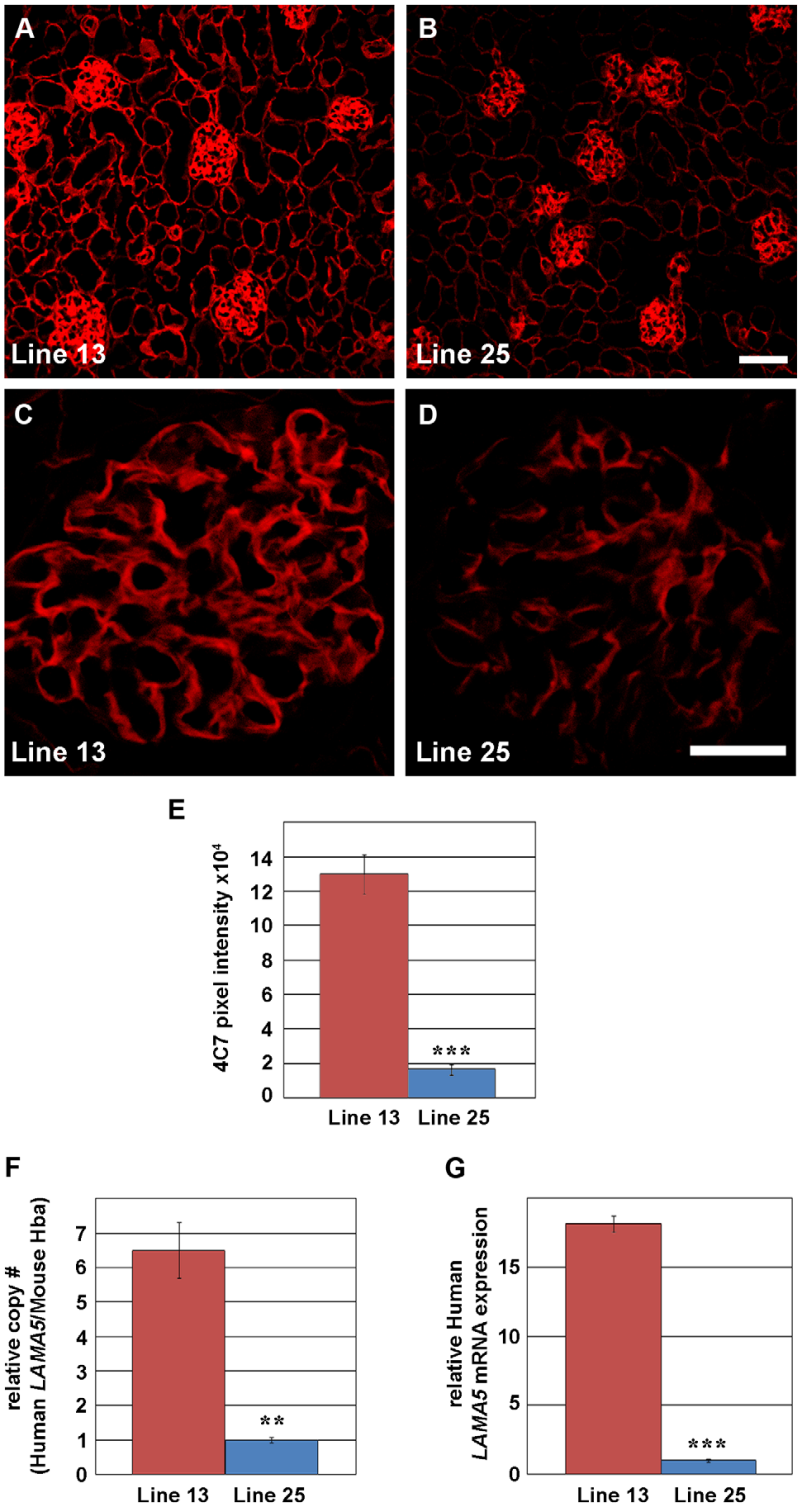
Characterization of two different human *LAMA5* BAC transgenic lines. A–D: Fresh frozen kidney sections labeled with mouse anti-human laminin α5 antibody, followed by anti-mouse IgG conjugated to Alexa Fluor-594. Sections from Line 13 and Line 25 were imaged by routine immunofluorescence microscopy (A and B), and by scanning confocal microscopy (C and D), using the same exposure settings. Linear basement membrane labeling of anti-human laminin α5 is seen in both line 13 (A and C) and 25 (B and D), but fluorescent signal appears brighter in Line 13. Magnification (A and B): 200×; scale bar = 50 µm. Magnification (C and D): 630×; scale bar = 20 µm. E: Quantification of glomerular immunofluorescence intensities shows significantly higher expression of human laminin α5 in GBMs of Line 13 mice, *** p<0.0001. F: Relative transgene copy number estimates were made using cycle threshold from quantitative real time PCR of human *LAMA5* genomic primers normalized to thresholds obtained with mouse hemoglobin A (Hba) primers. Compared to Line 25, Line 13 has more than 6 times as many copies of *LAMA5*, **p = 0.0013. G: Whole kidney total RNA from Line 13 or Line 25 (n = 3 samples per line) was amplified with human *LAMA5* intron-spanning primers, relative to PPIA (cyclophilin). Considerably more *LAMA5* mRNA is detected in Line 13, *** p<0.0001.

Although basement membranes in multiple organs from BAC transgenics were positive for human laminin α5, the intensity of immunofluorescence signals varied between the two different transgenic lines. For example, kidney glomerular and tubular basement membrane immunofluorescence in Line 13 appeared to be more intense than in Line 25 (Figs. 3A–D). When glomerular immunolabeling profiles were quantified by pixel intensity measurements of confocal microscope images, Line 13 glomeruli were more than 6 fold brighter than those in Line 25 (Fig. 3E). We interpreted the increased immunofluorescence signals in Line 13 to signify more human laminin α5 protein deposition.

To estimate relative gene copy number in the human BAC transgenics, a standard curve was amplified from human clone RP11-1023E23 DNA in 2-fold serial dilution (representing from 1 to 128 copies) spiked into 5 nanograms of mouse genomic DNA. Genomic liver DNA was purified from N2 progeny of Lines 13 and 25 and amplified using human-specific *LAMA5* primers, as well as mouse hemoglobin alpha (*Hba*) primers as a control (Table S1). Line 13 was estimated to have 6 times as many copies as line 25 (Fig. 3F). Similarly, mRNA expression levels were dramatically different between line 13 and 25, with line 13 expressing approximately 18 fold greater *LAMA5* message than Line 25 (Fig. 3G). These quantitative results suggest that the higher immunofluorescence signal seen in line 13 glomeruli was likely due to the higher *LAMA5* copy number and gene expression.

### Normal kidney function in human *LAMA5* BAC transgenics

In both transgenic lines, integration of the human *LAMA5* BAC into the mouse genome did not result in any overt phenotype for more than 8 months after birth. Heterozygous transgenic animals were active and fertile. Urine obtained from transgenic human *LAMA5* BAC mice at 4, 8, 20 and 24 weeks of age was separated by polyacrylamide gel electrophoresis (PAGE) alongside albumin standards, and stained with Coomassie Blue. There was no evidence of urinary albumin by PAGE in any of these samples (not shown). For further verification, albumin was quantified in urine from a total of 8 wildtype and 12 Line 13 transgenic mice at 4 weeks of age using a mouse albumin enzyme-linked immunosorbent assay. There were no significant differences in urinary albumin (wildtype = 6.7 µg/mL, BAC transgenic = 11.7 µg/mL, p = 0.111). Albumin levels quantified by enzyme-linked immunosorbent assay were also within normal limits in urine from transgenic mice at 8 weeks and 20 weeks of age. There were also no differences in blood urea nitrogen levels at 8 weeks of age (wildtype = 37.4 mg/dL, BAC transgenic = 36.5 mg/dL, p = 0.465).

**Figure 4:**
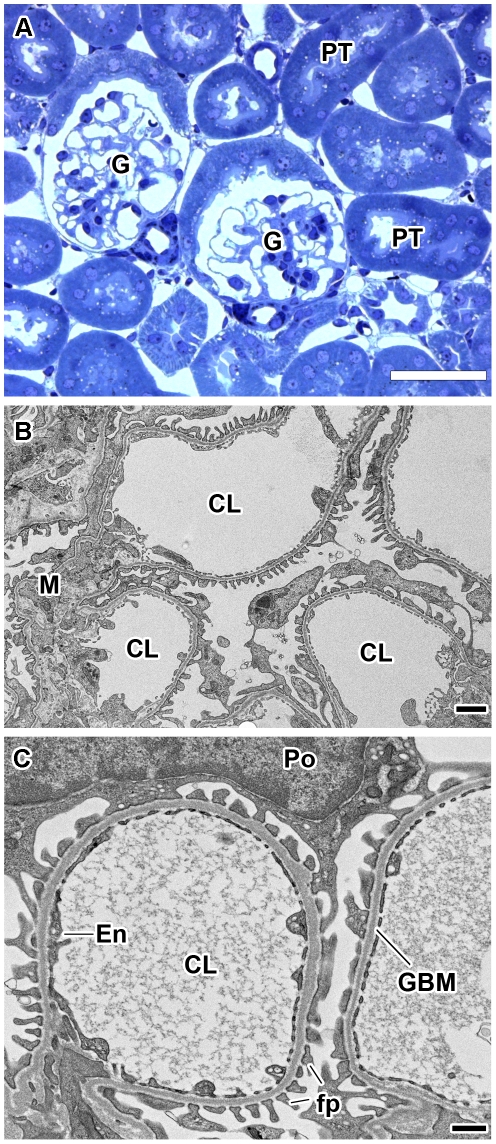
Normal kidney histology and glomerular capillary ultrastructure in Line 13 human *LAMA5* BAC transgenics. A: Semithin kidney section from an 8 week old transgenic stained with Toluidine Blue. Profiles of glomeruli (G) and proximal tubules (PT) are normal and there is no evidence of fibrosis or other defects. Magnification: 400×; scale bar = 50 µm. B: Electron micrograph of glomerular capillary loops from a human *LAMA5* BAC transgenic mouse showing normal mesangial (M) architecture and open capillary lumens (CL). Magnification: 7,000×; scale bar = 500 nm. C: Higher power view of glomerular capillary loops showing fenestrated endothelium (En), and normal, interdigitating foot processes (fp). The glomerular basement membrane (GBM) also appears to be of normal density and width. CL: capillary lumen, Po: Podocyte cell body. Magnification: 13,500×; scale bar = 500 nm.

Consistent with the renal function data, normal kidney histology was seen in transgenic human *LAMA5* BAC samples ranging in age from 3 days to 8 weeks old (Fig. 4A). The ultrastructure of glomeruli from 8 week old Line 13 transgenic mice also appeared entirely normal and displayed open capillary loops, fenestrated endothelium, GBMs of uniform thickness and density, and regular podocyte foot process registration (Figs. 4B and 4C). Similarly, there were no structural defects observed in kidney tubules or vasculature.

**Figure 5:**
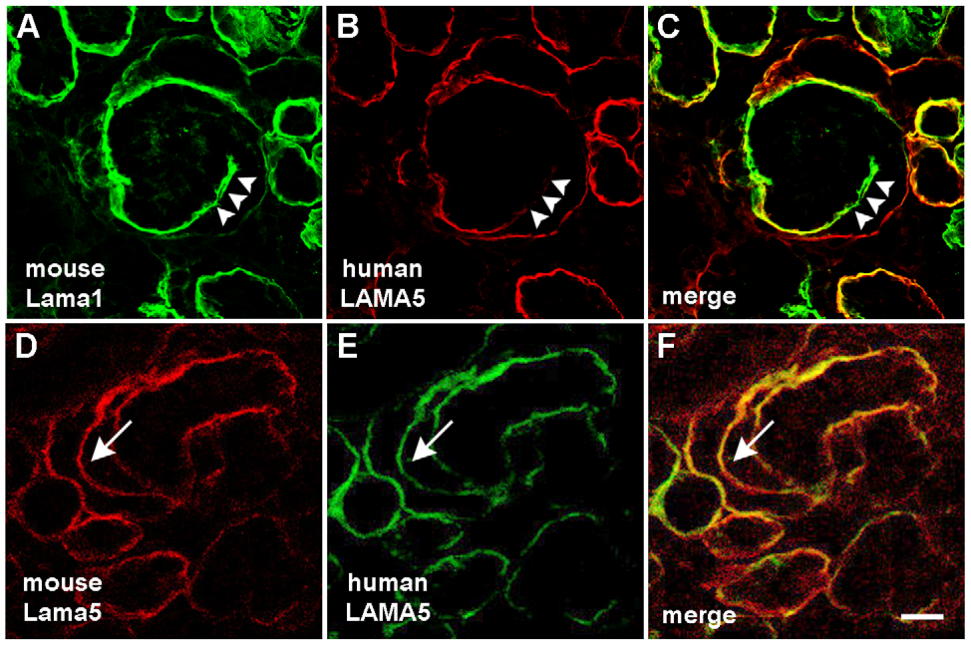
Human *LAMA5* expression occurs temporally correctly and coordinately with expression of mouse *Lama5*. A–C: Early GBM in vascular cleft of a comma-shaped nephric figure in neonatal human *LAMA5* transgenic immunolabeled with anti-mouse laminin α1 (A, green) and anti-human laminin α5 (B, red). At this early stage of glomerular development, the nascent GBM within the vascular cleft (arrowheads) contains predominantly the laminin α1 isoform with only trace amounts of laminin α5 present. D–F: At a slightly later stage of glomerular development, linear deposition of both mouse and human laminin α5 is evident in the GBM (arrow). Digitally merged images in F show colocalization of mouse and human laminin α5 in the same GBM (arrow). Magnification: 250×; scale bar = 20 µm.

We next asked whether expression of human laminin α5 protein in developing glomeruli followed the same temporal and spatial patterns observed for the intrinsic mouse protein. During normal glomerulogenesis, laminins containing the α5 chain (LN- 511 and LN-521) are first detected in the vascular clefts of comma- and S-shaped nephrons, where they replace the LN-111 isoform [14]. Subsequently, LN-521 is the only laminin isoform deposited into the GBM during glomerular maturation [13], [14]. To determine if the normal sequence of laminin α5 synthesis was occurring in the human *LAMA5* BAC transgenics, kidney sections from Line 13 newborn mice underwent double immunolabeling with anti-mouse laminin α1 and anti-human laminin α5 IgGs. As seen in Figure 5A, developing GBM within the vascular cleft of comma-shaped nephric figures contained mainly the endogenous mouse laminin α1, with only trace amounts of human laminin α5 of BAC origin (Figs. 5A–C arrowheads). At slightly later stages of glomerular development (S-shaped), more laminin α5 of both mouse and BAC origin was evident in the forming GBM (Figs. 5D–F, arrows). This signifies that the developmental expression of human *LAMA5* paralleled that for its murine homolog, and that both mouse and human laminin α5 chains were deposited concurrently within the same basement membrane segments.

**Figure 6:**
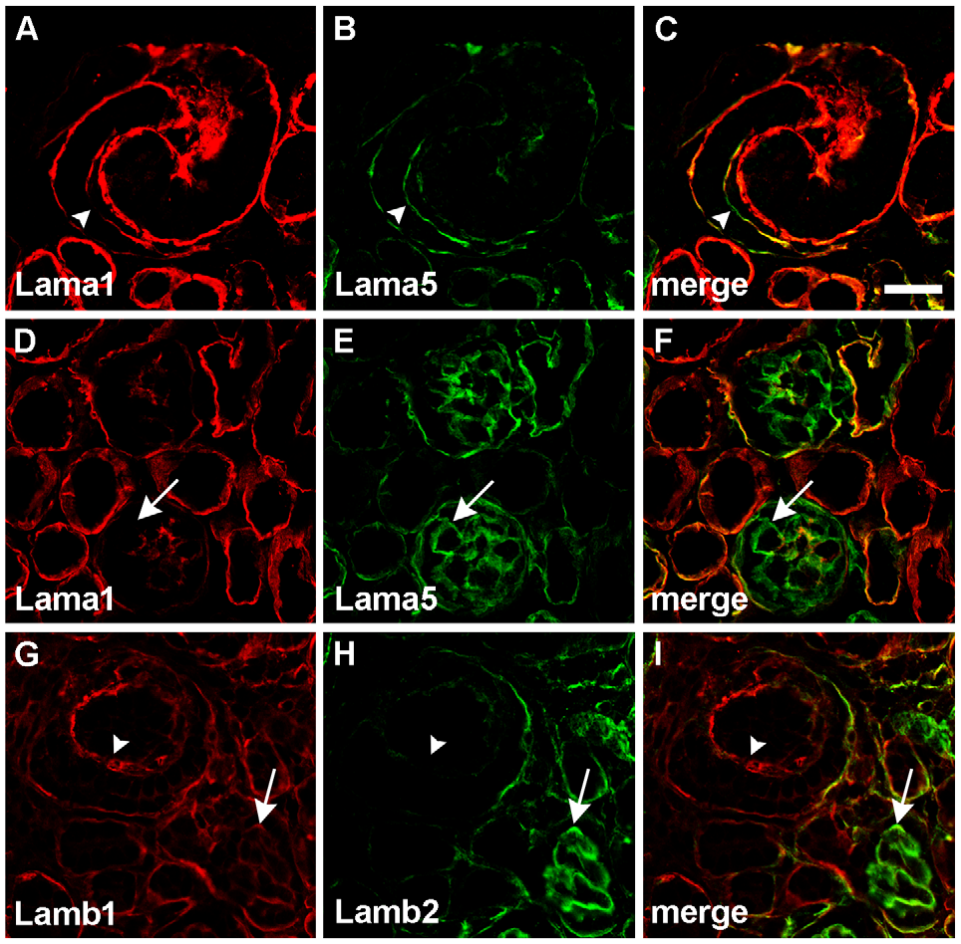
Expression of human *LAMA5* does not alter timing of mouse laminin isoform substitution. A–C: S-shaped nephric figure immunolabled for mouse laminin α1 (A, red) and mouse laminin α5 (B, green). Deposition of laminin α1 declines markedly (arrowhead) as laminin α5 appears. D–F: By the time glomeruli reach the capillary loop stage, laminin α1 is seen only in mesangial regions whereas laminin α5 occurs in loop GBMs (arrow). G–I: The normal laminin β1–β2 switch occurs somewhat slower than that for laminin α1–α5. G: Laminin β1 can be seen in vascular clefts (arrowhead) as well as in capillary loop stage GBMs (arrow). H: Trace amounts of laminin β2 are seen in vascular clefts and it becomes much more abundant in GBMs of capillary loop stage glomeruli (arrow). Magnification: 400×; scale bar = 20 µm.

We also wondered whether the expression of human *LAMA*5 might have affected the temporal isoform switching schedule of endogenous mouse laminin [13]–[14]. As shown in Figures 6A–C, early GBMs of S-shaped nephric figures contained both laminin α1 and α5, but at the capillary loop stage of glomerular development and thereafter, GBMs contained laminin α5 exclusively (Figs. 6D–F). The laminin β1–β2 isoform switch also occurred properly and on time (Figs. 6G–I).

**Figure 7:**
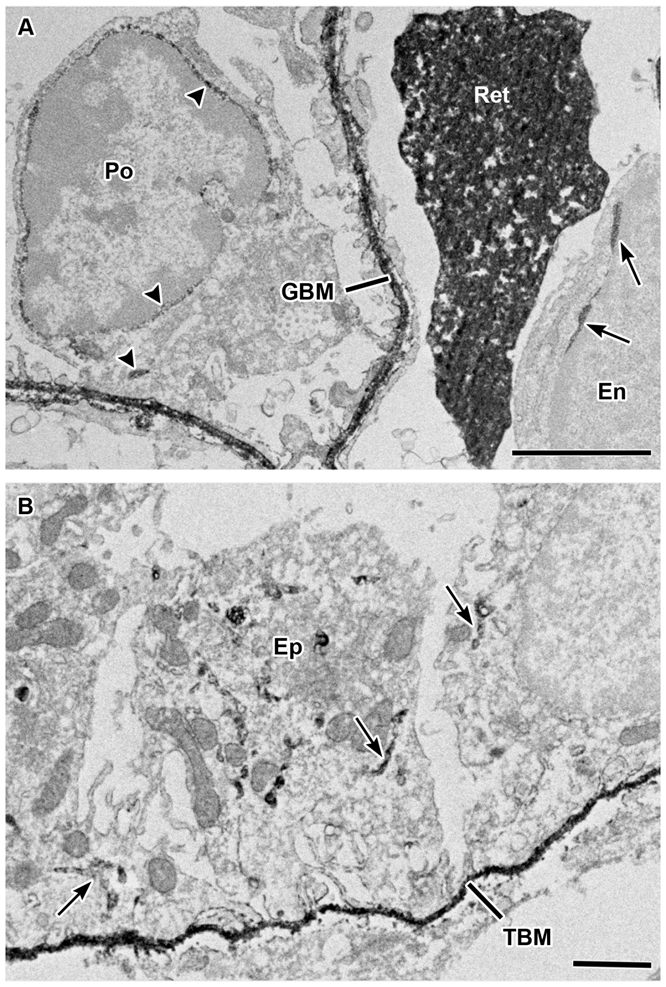
Post-fixation immunoperoxidase electron microscopy of newborn *LAMA5* transgenic kidney showing cellular origins of human laminin α5. A: Lightly fixed kidney sections were sequentially incubated with mouse anti-human laminin α5 IgG and then anti-mouse IgG conjugated to horseradish peroxidase. Tissue was then processed for peroxidase histochemistry and electron microscopy as described in Materials and Methods. Peroxidase reaction product, signifying anti-human laminin α5 IgG, is observed within biosynthetic organelles of glomerular endothelial cell (En) (arrows) and podocyte (Po) (arrowheads), as well as in the GBM. Ret: reticulocyte. Magnification: 13,000×. Scale bar = 2 µm. B: Human laminin α5 is also observed within biosynthetic organelles of tubular epithelial cells (Ep) (arrows) and in the tubular basement membrane (TBM). Magnification: 7,000×; scale bar = 2 µm.

To identify cellular sites of biosynthesis for human laminin α5, sections of lightly fixed BAC transgenic kidneys from 3 day old mice were treated with anti-human laminin α5 IgG and processed for immunoperoxidase electron microscopy. In glomeruli, peroxidase reaction product was seen intracellularly within endothelial cells and podocytes (Fig. 7A), which have previously been shown to be origins of mouse laminin α5 in developing glomeruli [16]. Similarly, intracellular vesicles within tubular epithelial cells were also labeled with anti-human laminin α5 in developing kidneys of BAC transgenics (Fig. 7B).

### Human laminin α5 protein binds mouse β1 and γ1 chains

**Figure 8:**
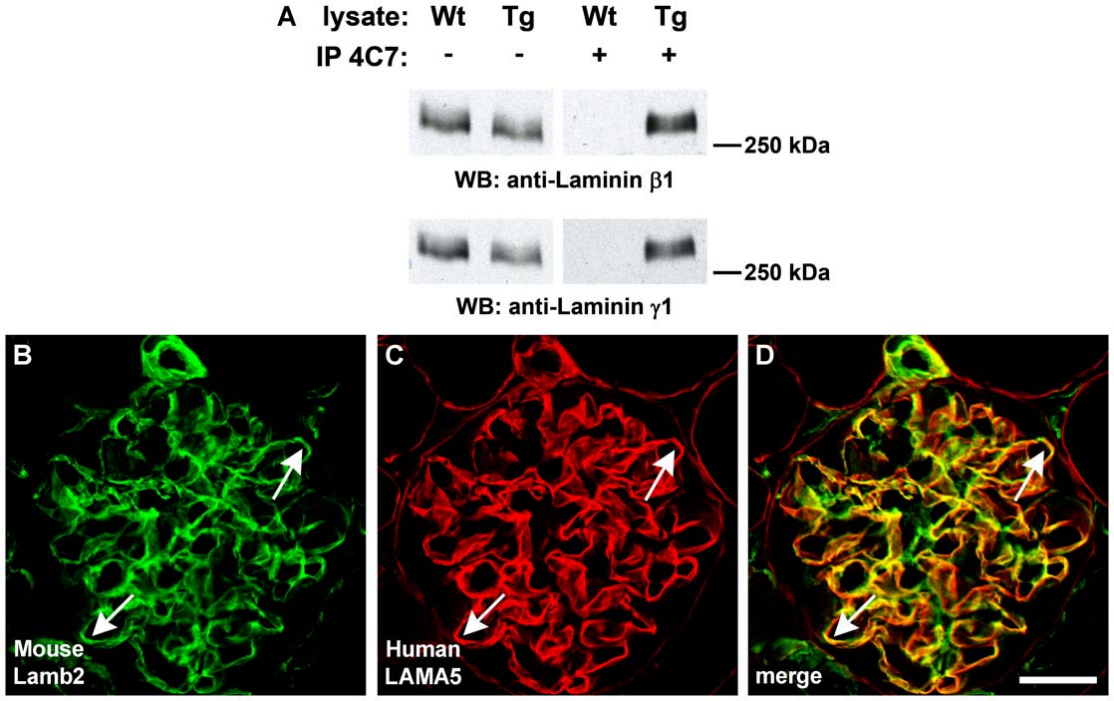
Human laminin α5 forms heterotrimers with mouse β1 and γ1 chains and co-localizes with mouse laminin β2 in GBMs. A: Postnatal day 2 kidneys were harvested from wildtype (Wt) or human *LAMA5* BAC transgenic littermates (Tg). Lysates were incubated with anti-human *LAMA5* antibody 4C7, and recovered with protein G beads (+). Western blotting with chain-specific anti-laminin β1 (top blot) or anti-laminin γ1 (bottom blot) shows that both wildtype and transgenic lysates contain laminin β1 and laminin γ1. Lysates from wildtype kidneys immunoprecipitated with anti-human laminin α5 4C7 antibody do not contain mouse laminin β1 or γ1, but both chains are present in immunoprecipitates from transgenic kidney. B–D: Double label immunofluorescence microscopy of fully mature *LAMA5* transgenic glomeruli shows widespread co-localization of mouse laminin β2 and human laminin α5 in GBMs (arrows). Magnification: 600×; scale bar = 20 µm.

Extraction of proteins from intact basement membranes has been notoriously difficult, making biochemical analyses of these matrices challenging. However, because we detected human laminin α5 protein within the intracellular biosynthetic pathways of glomerular endothelial cells and podocytes, as well as tubule epithelial cells in developing kidney, we reasoned that we should be able to solubilize intracellular laminin and determine whether it contained a heterotrimer of human and mouse chains. For these experiments, anti-human laminin α5 IgG was used to immunoprecipitate laminin from cell lysates of 2 day old BAC transgenic and wildtype kidneys. When immunoprecipitates from BAC transgenics were probed on Western blots with anti-mouse laminin β1 and γ1 chains, both were shown to be present (Fig. 8A). Although these immunoprecipitation experiments with whole kidney lysates could not define which nephron segment(s) contained human laminin α5-mouse β1-mouse γ1 heterotrimers, earlier evidence shows that developing GBMs of immature glomeruli contain LN-511, and this same laminin isoform is particularly abundant in immature and mature tubular basement membranes [14], [34]. In contrast to these findings from BAC transgenic lysates, kidney lysates from wildtype mice that eluted from anti-human laminin α5 were negative (Fig. 8A). Cell lysates from both wildtype and BAC transgenic kidneys that were not subjected to anti-human immunoprecipitation also contained laminin β1 and γ1 chains, as expected (Fig. 8A).

As mentioned earlier, the only laminin isoform in fully mature GBM is LN-521. To determine whether human laminin α5-mouse β2-mouse γ1 heterotrimers were also present in developing transgenic kidney lysates, anti-human laminin α5 immunoprecipitates were probed on Western blots with anti-mouse laminin β2. The results from these experiments thus far were negative. However, glomeruli represent only a small fraction of total kidney mass, and our negative result could have been due to insufficient sample within the total kidney lysate contributed specifically by maturing glomeruli. Because we saw what appeared to be large amounts of human laminin α5 in developing and mature glomeruli (Figs. 1, 3, and 5) we think that it is probable that human laminin α5-mouse β2-mouse γ1 heterotrimers are present within GBMs of the BAC transgenics. Further evidence in support of this came from double label immunofluorescence microscopy of adult transgenic glomeruli where, in most capillary loops, there was linear co-localization of anti-mouse laminin β2 and anti-human laminin α5 antibodies within GBMs (Figs. 8B–D).

### Expression of human *LAMA5* suppresses mouse *Lama5*

**Figure 9:**
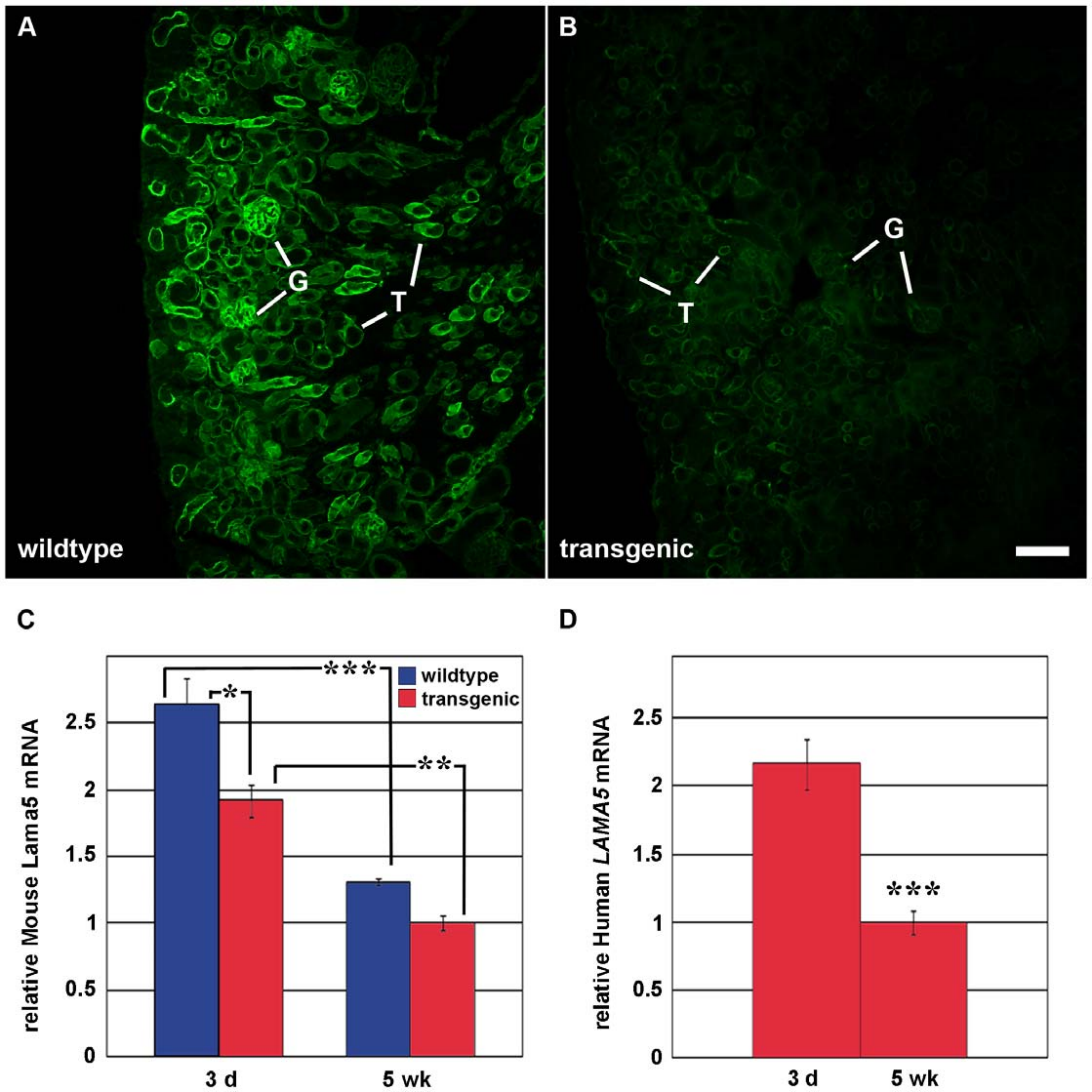
Human *LAMA5* BAC transgenics express less mouse laminin α5 protein and *Lama5* mRNA. A–B: Wildtype (A) and Line 13 *LAMA5* BAC transgenic (B) littermate kidneys were harvested and frozen sections were labeled with anti-mouse laminin α5. Digital images were captured using same exposure parameters. Compared to wildtype (A), note the marked reduction in immunolabeling for mouse laminin α5 in the transgenics (B). G: glomeruli; T: tubules. Magnification: 80×; scale bar = 100 µm. C: Total kidney RNAs from 3 day- (3 d) and 5 week-old (5 wk) wildtype (blue) and Line 13 transgenic (red) mice were amplified using mouse *Lama5* primers normalized to PPIA (cyclophilin). Significantly less mouse *Lama5* mRNA expression was seen in 3-day old *LAMA5* transgenic mice than in 3-day old wildtypes. Compared to 3-day olds, there was significantly less *Lama5* expression at 5 weeks for both wildtypes and transgenics. ANOVA, * p<0.05, ** p<0.01, *** p<0.001. D: Total kidney RNA from 3 day- and 5 week-old Line 13 human *LAMA5* BAC transgenics were amplified using human *LAMA5* primers. Compared to the 3-day old time point, the fold reduction in expression of human *LAMA5* mRNA at 5 weeks was similar to that seen for native mouse *Lama5* (C).

We next wondered whether the expression of apparently abundant human laminin α5 in Line 13 kidney affected expression of native mouse laminin. For these experiments, frozen sections from newborn wildtype and Line 13 BAC transgenic kidneys were labeled with anti-mouse laminin α5 and examined by immunofluorescence microscopy. In sections from wildtype kidney, bright linear immunolabeling for mouse laminin α5 was seen throughout glomerular and tubular basement membranes (Fig. 9A). By comparison, however, sections of human *LAMA5* BAC transgenic kidney showed an obvious and marked reduction in intensity of immunolabel for mouse laminin α5 (Fig. 9B), and glomeruli and tubules were equally affected. An earlier study in which doxycycline-inducible human *LAMA5* is expressed specifically in podocytes also noted what appeared to be a reduction of mouse laminin α5 within the GBM [29], but this observation was not pursued further.

To begin to investigate mechanisms accounting for the reduction in mouse laminin α5 protein, RNA was isolated from kidneys of 3 day old and 5 week old wildtype and Line 13 transgenic mice. Quantitative RT-PCR using mouse-specific primers showed that relative *Lama5* mRNA levels were significantly higher in 3 day old kidneys than at 5 weeks of age, and this was true for both wildtype and human *LAMA5* BAC transgenics (Fig. 9C). Similarly, the relative amount of *LAMA5* mRNA in 3 day old BAC transgenics was significantly higher than at 5 weeks, and the fold decrease at 5 weeks was approximately the same as that for mouse *Lama5* (Fig. 9D). We interpret these findings to reflect a burst of basement membrane assembly that accompanies the rapid induction and elongation of nephrons that occurs during kidney development. This would require more message for basement membrane proteins in kidneys from 3 day olds than at 5 weeks of age, when kidneys have reached their nearly mature size and basement membrane assembly has essentially concluded.

Our results from 3 day old, Line 13 *LAMA5* BAC transgenic kidneys also showed less mouse *Lama5* mRNA when compared to wildtype (Fig. 9C). Although the reduction in mouse *Lama5* mRNA in transgenics was not as striking as what was seen at the protein level by immunofluorescence, it nevertheless was statistically significant (Fig. 9C). In addition, the loss was specific for *Lama5* as qRT-PCR of Line 13 and wildtype kidneys showed no differences in expression of *Lama1*, *Lamb1*, *Lamc1*, or *Col4a1* mRNAs. Moreover, there were no differences in *Lama5* message between wildtype mice and Line 25 BAC transgenics, which expressed substantially lower amounts of human *LAMA5* than Line 13, and the intensity of anti-mouse laminin α5 immunolabeling also appeared closely similar between Line 25 and wildtypes.

What mechanism(s) could account for the suppression of mouse *Lama5* mRNA and reduced mouse laminin α5 protein deposition in the human *LAMA5* BAC transgenics? Clearly, the transgenics transcribed both the human *LAMA5* and mouse *Lama5* genes, and, as shown in Figures 5D–F, laminin protein from both species was deposited in the same kidney basement membranes. Perhaps the reduction in mouse *Lama5* message seen in the *LAMA5* BAC transgenics was the result of a feedback mechanism in which the total amount of laminin α5 chain protein (mouse and human) somehow subdued the tempo of *Lama5* gene transcription. Alternatively, there may have been competition between the human *LAMA5* and mouse *Lama5* genes for the same transcription factor(s). On the other hand, there may have been loss of *Lama5* mRNA stability or other post-transcriptional changes in the presence of excess *LAMA5* message.

Earlier work in cell culture has shown that the laminin α1 chain can be secreted separately from its β1 and γ1 subunit partners, but that most of the secreted α chain monomer undergoes proteolytic cleavage [35]. In contrast, the laminin β1 and γ1 subunits are not secreted in the absence of α1, and accumulate intracellularly [35]. As already mentioned, qRT-PCR of Line 13 and wildtype kidney samples showed that there were no differences in relative message abundance for *Lamb1* or *Lamc1*, which encode laminin β1 and γ1 chains, respectively, and which we showed were binding partners for human laminin α5 (Fig. 8). Perhaps the secretion of a stable human laminin α5-mouse β1-mouse γ1 chimeric heterotrimer, and entirely mouse heterotrimers, was rate-limited by the amounts of laminin β and γ chains available for heterotrimerization. Nevertheless, how the abundance of laminin α5 protein could autoregulate *Lama5* gene transcription is not at all clear, but this could be an important control mechanism that becomes defective in fibrotic conditions where there is overproduction of basement membrane protein.

In summary, we have generated transgenic lines of mice that express human *LAMA5* in temporally and spatially correct contexts within kidney, indicating that the appropriate genetic control elements are present. Unexpectedly, a transgenic line expressing the highest amounts of human laminin α5 suppressed mouse *Lama5* mRNA and mouse protein deposition. These transgenics may prove useful for understanding regulation of laminin gene expression and provide new clues regarding mechanisms of basement membrane assembly.

## Supporting Information

Table S1 Primers complementary to human (capitalized gene symbols) and mouse were designed using the indicated accession numbers as templates, and each pair was given a unique primer designation. Primer sequence and length in basepairs is also shown.
(DOC)
