# Supplementary material for: Transgenic Expression of Human LAMA5 Suppresses Murine Lama5 mRNA and Laminin α5 Protein Deposition
Source: PLoS One. 2011 Sep 7;6(9):e23926. doi: 10.1371/journal.pone.0023926 (PMC3168496; doi:10.1371/journal.pone.0023926)
Supplement: Table S1 — Primers complementary to human (capitalized gene symbols) and mouse were designed using the indicated accession numbers as templates, and each pair was given a unique primer designation. Primer sequence and length in basepairs is also shown. (DOC) [file pone.0023926.s001.doc]

**Supporting Information Table S1. Primers Used for Human BAC Transgenic Analysis**

| **Gene Symbol**  **Accession** | **Primer**  **designation** | **Primer sequence** | **Product length (bp)** |
| --- | --- | --- | --- |
| ADRM1  Hu Chr 20 60840359-61029865 | ADRM1For | 5’- TACATTCAGCAGACGGACGA -3’ | 1795 |
|  | ADRM1Rev | 5’- ACACGTTCTACACCAGCAGG -3’ |  |
| C20orf151  Hu Chr 20 60840359-61029865 | ORF151For | 5’- GCAACACTGGCTCTCACCTA -3’ | 1846 |
|  | ORF151Rev | 5’- AAGGCTGAAGTGCTGAGACC -3’ |  |
| CABLES2  Hu Chr 20 60840359-61029865 | CABLES2For | 5’- AGCGCTGGACCATGCAGAAT -3’ | 1790 |
|  | CABLES2Rev | 5’- TACATCGCTCCAGCCTCGGT -3’ |  |
| Col4a1  [NM_009931.1](https://qpcr2.probefinder.com/MainServlet.do;jsessionid=F1EF61E8BC23B61E712A91EC238BB4AD#%23) | Col4a1S1 For | 5’-CTGGCACAAAAGGGACGAG-3’ | 238 |
|  | Col4a1S1 Rev | 5’-ACGTGGCCGAGAATTTCACC-3’ |  |
| Col4a2  NM_009932.2 | Col4a2S2 For | 5’- TGCTACCCGGAGAAAGGAG -3’ | 106 |
|  | Col4a2S2 Rev | 5’- CTTTGCGGCCCTGTAGTCC -3’ |  |
| Dag1  NM_0100170.1 | Dag1P99For | 5’- ctgctgctgctccctttc -3’ | 95 |
|  | Dag1P99Rev | 5’- gcagtgttgaaaaccttatcttcc -3’ |  |
| Hba-a1  NM_008218.2 | Hba-a1 For | 5’- actaacttcttcccaaactgccatca -3’ | 221 |
|  | Hba-a1 Rev | 5’- aagggctgtcctccaggcagggtgg -3’ |  |
| Itga3  NM_013565.2 | Itga3P17For | 5’- tcaacatggagaacaagacca -3’ | 90 |
|  | Itga3P17Rev | 5’- ccaaccacagctcaatctca -3’ |  |
| Itgb1  NM_010578.1 | Itgb1P19For | 5’- atgcaggttgcggtttgt -3’ | 73 |
|  | Itgb1P19Rev | 5’- catccgtggaaaacaccag -3’ |  |
| Lama1  NM_008480.2 | Lama1P99 For | 5’- cgcaggacactcctgtca -3’ | 64 |
|  | Lama1P99 Rev | 5’- accactttctgggagcttttc -3’ |  |
| Lama5  NM_001081171.2 | Lama5P2 For | 5’-ACCCAAGGACCCACCTGTAG-3’ | 169 |
|  | Lama5P2 Rev | 5’-TCATGTGTGCGTAGCCTCTC-3’ |  |
| LAMA5  NM_005560.3 | LAMA5P32H.s. For | 5’- Cctcgtcctccaatgacac -3’ | 70 |
|  | LAMA5P32H.s.Rev | 5’- gcgctgcagtcacaattc -3’ |  |
| LAMA5  Hu Chr 20 60840359-61029865 | LAMA5 intron2For | 5’- CTGGACACGAAGGACTTGGT -3’ | 938 |
|  | LAMA5intron2Rev | 5’- GCTCTGCAAGTTCAGGAACC -3’ |  |
| Lamb1-1 NM_008482.2 | Lamb1-1P66 For | 5’-ggcaaactgcaaagtctcg-3’ | 61 |
|  | Lamb1-1P66 Rev | 5’-ctggaggtgttccacaggtc-3’ |  |
| Lamb2  NM_008483.2 | Lamb2S2 For | 5’- GTGTGGCTTGCATAGCCCT-3’ | 122 |
|  | Lamb2S2 Rev | 5’- TCCGATGACTATTTGGGTTGTCT-3’ |  |
| Lamc1  NM_010683 | Lamc1-1For | 5’- Tgccggagtttgttaatgcc -3’ | 185 |
|  | Lamc1-1Rev | 5’- ctggttgttgtagtcggtcag -3’ |  |
| OSBPL2  Hu Chr 20 60840359-61029865 | OSBPL2For | 5’- TGTGGACTGTGGCCTCTTCT -3’ | 1873 |
|  | OSBPL2Rev | 5’- AGAGCCAGACTCCGTCTCAA -3’ |  |
| Ppia  NM_008907 | cyclophilin For | 5’-CAGACGCCACTGTCGCTTT-3’ | 132 |
|  | cyclophilin Rev | 5’-TGTCTTTGGAACTTTGTCTGCAA-3’ |  |
